# When addressing resources is not enough: lessons learned from a respectful maternal and neonatal care provider training intervention evaluation in Kenya and Tanzania

**DOI:** 10.1186/s12884-024-06555-3

**Published:** 2024-05-14

**Authors:** Matthea Roemer, Uri Eduardo Ramírez Pasos, Inviolata Wanyama, Esther Lubambi, Angela Argenziano, Patricia Lledo Weber

**Affiliations:** 1 MSI Reproductive Choices, 1 Conway Street, Fitzrovia, London, W1t 6LP UK; 2 Marie Stopes Kenya, Nairobi, Kenya; 3 Marie Stopes Tanzania, Dar es Salaam, Tanzania

**Keywords:** Respectful maternity care, Respectful neonatal care, Obstetric violence, Values and beliefs, Providers’ attitudes, Positive pregnancy experience, Patient-centred care

## Abstract

**Background:**

Respectful Maternal and Neonatal Care (RMNC) maintains and respects a pregnant person’s dignity, privacy, informed choice, and confidentiality free from harm and mistreatment. It strives for a positive pregnancy and post-pregnancy care experiences for pregnant people and their families, avoiding any form of obstetric violence. Though RMNC is now widely accepted as a priority in obstetric care, there is a gap in resources and support tools for healthcare wproviders to clearly understand the issue and change long-established practices such as non-humanized caesarean sections. MSI Reproductive Choices (MSI) manages 31 maternities across 7 countries with a zero-tolerance approach towards disrespectful maternity care and obstetric violence. MSI developed and implemented a hybrid training package, which includes an online module and 1-day in-person workshop that allows healthcare providers to explore their beliefs and attitudes towards RMNC. It leverages methodologies used in Values-Clarification-Attitudes-Transformation (VCAT) workshops and behaviour change approaches.

**Methods:**

The impact of this training intervention was measured from the healthcare providers’ and patients’ perspectives. Patient experience of (dis)respectful care was collected from a cross-sectional survey of antenatal and postnatal patients attending MSI maternities in Kenya and Tanzania before and following the RMNC training intervention. Healthcare providers completed pre- and post-workshop surveys at day 1, 90 and 180 to measure any changes in their knowledge, attitudes and perception of intended behaviours regarding RMNC.

**Results:**

The results demonstrate that healthcare provider knowledge, attitudes and perceived RMNC practices can be improved with this training interventions. Patients also reported a more positive experience of their maternity care following the training.

**Conclusion:**

RMNC is a patient-centred care priority in all MSI maternities. The training bridges the gap in resources currently available to support changes in healthcare wproviders’ attitudes and behaviours towards provision of RMNC. Ensuring health system infrastructure supports compassionate obstetric care represents only the first step towards ensuring RMNC. The results from the evaluation of this RMNC provider training intervention demonstrates how healthcare provider knowledge and attitudes may represent a bottleneck to ensuring RMNC that can be overcome using VCAT and behaviour change approaches.

**Supplementary Information:**

The online version contains supplementary material available at 10.1186/s12884-024-06555-3.

## Background

Until very recently, the sole marker of high quality maternal and newborn care was the absence of maternal, foetal, and neonatal deaths [1]. The focus on saving the mother’s and newborn’s life overshadowed the pregnant persons’^1^ and newborn’s experience during pregnancy and relegated this experience to a secondary place. There was (and still is in cases) an underlying assumption that both saving lives and providing a positive experience are mutually exclusive and one had to be prioritized over the other [1, 2].

The term “childbirth abuse”, also known as “obstetric violence”, refers to the violation of a person’s rights during childbirth. The terms can be interchangeable. They describe a broad range of behaviours and attitudes that dehumanize, pathologize, and abuse individuals during reproductive processes, especially childbirth. Verbal and physical abuse^2^ are the most obvious types of violence and the ones most often associated with the term obstetric violence. However, many other day-to-day routine obstetric practices are disrespectful but continue to be taught and practised across generations of obstetric healthcare providers. Some examples are failure to offer and provide pain management options, refusal to allow a birth companion, refusal to adopt unharmful cultural preferences during the delivery (e.g., certain positions during labour and delivery), and separation of mother and baby for non-medical reasons.

Providing a positive experience during pregnancy and childbirth means avoiding any form of mistreatment and abuse, promoting respectful obstetric practices and fostering interventions such as skin-to-skin, labour companions and different birthing positions with an overall person-centred approach.

Such respectful maternal and newborn care can decrease perinatal mental health problems and improves mother-baby bonding and breastfeeding. Newborns who are not separated from their mothers for non-clinical reasons and receive skin-to-skin care, experience less respiratory distress and admissions to specialized care, manage thermoregulation better and have fewer feeding problems [3]. Respectful and supportive care during labour also decreases caesarean section rates and pain perception during labour [3]. Therefore, a disrespectful and negative experience during pregnancy is not only a human rights violation [1, 2] but also has detrimental consequences to the health of the pregnant person and newborn on several levels.

From a public health point of view, the impact of obstetric violence and disrespectful care will effectively interfere with the efforts to promote safe deliveries in health facilities that allow access to skilled staff and safe referral [4]. Qualitative research on disrespect and abuse during childbirth in countries such as Ethiopia, Kenya, Mozambique, and Tanzania exposed concerning degrees of disrespect and abuse [5]. Additionally, disrespect and abuse during pregnancy and childbirth, may be more prevalent in underserved and vulnerable populations as it feeds from toxic gender and power dynamics, increasing inequities in maternal and newborn health across the globe [6, 7].

In 2014, WHO called for action, dialogue, research, and advocacy on respectful maternity care because “Every woman has the right to the highest attainable standard of health, which includes the right to dignified, respectful health care throughout pregnancy and childbirth, as well as the right to be free from violence and discrimination.“ [8].

Since then, WHO released several clinical guidelines on antenatal care, postnatal care and labour care that emphasise clinical and non-clinical aspects to foster respectful obstetric care and a positive experience [9–11]. The new labour care guidelines incorporates features of supportive care, and pain relief and aims to reduce unnecessary and hasty medical interventions [12, 13]. The WHO implementation toolkit offers insights into the complex range of contributing factors to disrespectful maternity care and how to assess and approach them from a health system and facility perspective [14].

In addition to the WHO guidelines, a recently released series of five papers explored in depth the complex myriad of contributing factors and proposed a range of high-level strategies to end childbirth mistreatment and improve respectful care [15–19]. The identification of the contributing factors to childbirth abuse opens the door to effective interventions at all healthcare levels. These factors include underlining power and gender dynamics, deficiencies in essential physical resources (e.g., inadequate supply chains, poor physical maternity conditions that do not facilitate respectful practices such as privacy or moving during labour), poor supervisory structures, insufficient staffing, and competencies, motivations and beliefs of the staff.

MSI Reproductive Choices (MSI) operates a multi-country network of maternities, with 31 maternities operational across 7 countries in low- and middle-income countries in 2023. Beginning in 2021 as part of routine quality assurance mechanisms, MSI conducts yearly external audits for each maternity, which incorporate an adapted version of the WHO basement assessment tool for implementation of respectful intrapartum and immediate postnatal care [14]. Through these audits, gaps in essential physical resources were identified and corrected. These gaps included adequate staffing ratios, physical conditions to improve patient flows, privacy and dignity as well as fostering a strong RMNC culture at facility, country, regional and organizational levels.

As a follow-up to these audits, additional guidance and protocols were developed to encourage, describe, and train staff members in respectful obstetric practices. In addition, materials were adapted in all maternities to be context-specific, including birth plans, counselling for birth companions and training on person-centred communication.

This was, however, only the beginning of change. Obstetric training for doctors and midwives is historically not focused on providing a positive experience but on saving the pregnant person’s and newborn’s life by any means. Supporting a sustained change in long-standing beliefs and attitudes represented the biggest challenge. Despite the availability of frameworks of care, standards, and tools for evaluating RMNC, we found that practical tools that deep dive into the beliefs and attitudes of maternity providers were not widely available.

In close collaboration with country teams in Uganda, Kenya, Tanzania, Ethiopia, Madagascar, and Bangladesh, MSI developed an intervention package to ensure that all providers were adequately supported in their journey towards providing respectful obstetric care to all our patients. The package includes a provider workshop based on behaviour change methodology and it includes a variety of strategies to sustain, in the long-term, the behaviour changes and the drive towards respectful care.

As part of the initial rollout of the RMNC package in Kenya and Tanzania, an interventional evaluation was completed to understand the feasibility of the implementation of the RMNC training toolkit, and any impact experienced by both providers and patients.

Specifically, the evaluation aimed to:

- Understand the feasibility of rolling out the training and other operational changes planned to support the systematic delivery of RMNC.
- Understand the level of provider satisfaction with the RMNC training, and resulting provider knowledge, attitudes towards RMNC, and perceived RMNC practices.
- Understand if providers are offering respectful care that preserves dignity, confidentiality, and choice, including providers respecting mother’s preferences and ensuring all patients have birth plans in place.
- Understand if obstetric violence is avoided and whether patients are more satisfied with the care following the rollout of the RMNC package.

## Methods

### Description of the intervention

The specific intervention under evaluation is the training toolkit developed to change specific beliefs and attitudes about RMNC relating to the respect for non-harmful contextual cultural beliefs and actions. The training toolkit is based on the established Values-Clarification-Attitudes-Transformation (VCAT) and behavioural change methodologies. These workshops encourage participants to explore their assumptions about abortion and examine their role in assuring people’s safe access to abortion care [20]. The types of exercises used in VCAT were specifically adapted for the prevention of obstetric violence.

The objective of the training toolkit is to support participants in identifying forms of obstetric violence in their day-to-day work and find solutions and alternatives for respectful practices. The target participants are first line obstetric providers (midwives, doctors). Still, it is strongly recommended to include managerial and support staff as well as crosscutting staff involved in the care of the mother and newborn, such as anaesthetists and paediatricians.

The toolkit is hybrid. The first module is online, can be completed in a short period of time (45–60 min), is available in different languages and does not require high technological literacy. The objective of this initial online module is to build up knowledge about respectful maternity care through stories, activities, and quizzes.

The in-person, 1 day (8 h) workshop is the second module and takes place after completion of the e-learning. The workshop trainers must have an obstetric clinical background to be able to guide the discussion and solutions of respectful obstetric practices, as well as have received the RMNC training and have undergone a Training of Trainers (ToT) session. The workshop includes 7 dynamic activities that build up on each other. It explores the concepts of bodily autonomy, power and gender dynamics through symbolic exercises, role plays, scenarios and group activities that allow teams to explore and challenge their beliefs and attitudes and identify and overcome non-RMNC behaviours in their day-to-day work.

The training toolkit has undergone several iteration cycles and is Continuing Professional Development (CPD) certified. The CPD Certification Service provides support, advice and recognised CPD accreditation for the Continuing Professional Development obligations and policies of professional bodies and academic institutes [21].

The evaluation of the intervention presented hereby includes the data from Kenya and Tanzania MSI obstetric programs where all fulltime maternity staff were trained, except for maternity leaves and part-time or locum staff, using this RMNC training package. Evaluation in MSI Ethiopia maternities is currently ongoing with results expected in early 2025.

### Study design

This study drew on multiple data sources with the aim of holistically capturing both patient and provider perspectives. This included a (a) pre-post cross-sectional remote interviewer-administered patient survey and (b) a self-administered survey with all RMNC training participants at 4-time points: immediately before the training, immediately following the training, approximately 90 days post training and again approximately 180 days post training.

In addition, routine services data including patient records data, clinical quality data, and patient feedback data were regularly monitored during the intervention implementation to inform iterative adaptations as needed.

This paper presents the results from the primary, quantitative data collected via the patient and provider surveys, responding to the latter 3 aims of the evaluation. However, routine monitoring of operational data and staff feedback indicates that it was practically feasible to rollout the RMNC training package and corresponding structural changes in the maternities.

### Study setting

Data collection was drawn from patients and providers from 2 maternities in Mombasa and Nairobi, Kenya and 1 maternity in Dar es Salaam, Tanzania. Data was collected between February 2023 and September 2023.

### Sampling approach

For the provider survey, all in-person RMNC workshop participants, which included all fulltime maternity staff, were invited to complete the provider survey. In Kenya, 22 maternity staff attended the training and 24 maternity staff attended the training in Tanzania. The potential participants were informed about the aim of the survey before the training commenced and were informed that their decision to participate in the survey (or not) would not affect their participation in the training. 83% (38/46) of providers (17 from Kenya and 21 from Tanzania) agreed to participate and completed the full survey at baseline (immediately pre-training and/or immediately post-training). For the 90-day and 180-day follow-up surveys, all training participants were contacted by e-mail or WhatsApp and invited to complete the survey. Follow-ups were completed a total of 3 times to encourage completion. A total of 17 participants completed the 90-day survey (17 from Kenya, 0 from Tanzania) and 43 participants (21 from Kenya, 22 from Tanzania) completed the 180-day survey. Considering the missing provider survey data from Tanzania, only the provider survey data from Kenya is presented in the results.

For the patient survey, all patients aged 18–49 who completed an antenatal care (ANC) visit, delivered, and/or completed a post-natal care (PNC) visit at one of the three [3] maternities in the 8 weeks prior to the in-person RMNC training or for 8 weeks approximately 6-months after the training were eligible for the survey. Patients were excluded if they were under the age of 18, did not have a record of consenting to be recontacted in the Electronic Health Records system, or did not deliver at one of the three [3] maternities (in the case of post-natal care patients).

A simple random sampling approach was used with each country as a unique stratum, applying the following formula and assumptions:

Sample size (n) = Z_a_^2^ pq / d^2^

where:

- Z_a_=1.96, corresponding to a confidence level of 95%.
- *p* = 50%, hypothesized % frequency of outcome factor in the population.
- q = 1- p.
- d = 10%, corresponding to the confidence limits as % of 100.

This gives the following minimum sample size: n = (1.96^2^ × 0.5 × 0.5)/0.10^2 =^ 97.

We then assumed a non-response rate of 30% and that the phone coverage would be 90% in Tanzania and 100% in Kenya based on MSI’s internal 2021 patient exit interview data. This gave us a final sample size of 139 patients to call in Tanzania and 127 patients to call in Kenya (See Table 1).

**Table 1:** Sample size estimates for patient survey

Country	Minimum sample (n)	Minimum sample with applied non-response rate	Minimum sample to call with applied phone coverage
Kenya	97	127	127
Tanzania	97	127	139

### Data collection

For the provider survey, a self-administered online (Microsoft Forms) questionnaire was administered in English to collect data on the participants’ sociodemographic, professional, and work-related characteristics, attitudes towards RMNC, and perceptions of their own and other providers’ likely behaviours around RMNC. The same questionnaire was used at the time points (immediately prior to the 1-day RMNC training, immediately following the 1-day training, 90 days post training and 180 days post training). However, for the immediate post-training survey, additional questions were asked to gather feedback on the in-person training.

An information sheet and consent statement were included at the start of each provider survey, including information on the purpose of the survey, how the data could be used, the risks and benefits to the participant, and that participation was fully voluntary and could be withdrawn at any time. All survey responses were fully anonymous, and this was made clear on the information page as well.

For the patient survey, an interviewer-led remote survey was conducted using computer-assisted personal interviewing (CAPI). A trained interviewer read out the questionnaire to patients over the phone while using an electronic data-capture system (KoboToolbox) on a tablet to record the participant’s responses. The interviews were conducted by MSI-employed contact centre agents following a full day of in-depth data collection training. Contact centre agents were well placed to administer the survey as they are already trained in conducting follow-up calls with patients and how to do so respectfully and confidentially, ensuring the patient has a private place to speak from and is comfortable with the call to continue. However, the data collection training still covered in detail the consent process for this survey to ensure the interviewers were clear on the process and comfortable following it.

Each eligible patient participant was called a maximum of three [3] times depending on their response to the initial calls. If a patient did not answer on the first attempt, this was recorded in an electronic call log. The interviewer would wait a minimum of 30 min before trying again. If they did not pick up on the 2nd call, the interviewer would wait until the next day to call again. If they still did not respond, this was logged as a non-response.

If the eligible patient did respond but noted that the time was inconvenient, this was recorded in the call log as well as the reported convenient call-back time. The interviewer would then call the patient back at that time. Once the survey was completed, this was recorded as well in the call log.

When a call was answered, the interviewer would begin by confirming that they had the correct potential interviewee on the line. Once confirmed and provided with verbal consent to continue the call and that they were in a comfortable place to do so, the interviewer would read out to them an information sheet, provide time for any questions, and seek verbal consent to continue the interview.

Once consent was provided, the questionnaire took 20–25 min to complete. The questionnaire covered questions on patient demographics, satisfaction with care, experience with overall care, experience of facility environment, and experience of respectful care. Two validated scales for measuring respectful care were included in the questionnaire: the Mothers on Respect (MOR) index and the Mistreatment Index (MIST) [22, 23]. The MOR index was developed through a participatory research process and has previously been validated with patient populations in the US and Canada. It is a 14-item scale that pregnant or postpartum persons can use to rate their level of comfort during a provider-patient interaction, willingness and comfort with asking questions, and perception of (dis)respect and discrimination when receiving care [24]. The MIST is a patient designed set of indicators that explores the seven dimensions of mistreatment according to Bohren typology [5] and can be used to identify any type of violence during childbirth. This index has previously been used in a US national study [23].

### Data analysis

For the provider survey data, descriptive statistics were computed to explore trends in agreement with the various statements around providers’ knowledge, attitudes, and behaviours towards RMNC at each survey timepoint. Significance testing was not run given the small sample sizes and missing data in Tanzania for two time points. It was also not possible to match observations across survey timepoints as no unique identifiers were captured in the survey to support anonymity.

For patient data, survey indicators were grouped into (1) MOR (2), MIST, and (3) Patient-centred care. MOR indicator values were added to obtain an overall score (14–84) in line with the approach described by Vedam et al. [24]. MIST indicators were collapsed into a binary indicator “Experienced mistreatment” and patient-centred care indicators were analysed individually to assess the level of agreement with each statement.

At baseline, descriptive statistics were run for all variables to assess the level of agreement with the patient-centred care indicators as well as the scores for the MOR, with a detailed breakdown analysis run to assess the mean score for each MOR category. This informed the approach to the endline analysis. For patient-centred care variables with response options of ‘Yes’, ‘No’, or ‘Don’t know’, those with fewer than 80% responding positively at baseline were included in the endline analysis tests for differences. Similarly, for patient-centred care variables with 5-point Likert scale response options from ‘Strongly Agree’ to ‘Strongly Disagree’, those with fewer than 90% ‘Strongly Agree’ at baseline were included in the endline analysis tests for differences (see appendix 3 for the complete question set). For the detailed analysis of the MOR score, all categories with a score of 5.5 or lower were assessed for significant differences at endline.

At endline, for the MOR scores and unique categories, differences between baseline and endline were assessed via an unpaired two-sample t-test. All categories with significant differences between baseline and endline are reported on in the results (see Appendix 2 for a complete table). For MIST indicators, the difference between baseline and endline was assessed via a chi-squared test. Differences between baseline and endline for the patient-centred care indicators were evaluated individually using chi-squared tests.

For the MOR category analysis, multiple testing was controlled by applying a Bonferroni correction to set a statistical significance at *p* < 0.0036. This was derived from an established *p* < 0.05 divided by 14 (the number of MOR categories). For all other hypothesis tests, statistical significance was set at *p* < 0.05.

Provider survey data were analysed using Stata version 15 and patient survey data were analysed using R/RStudio version 2023.9.1.494 (patient survey data).

### Ethical approval

This protocol was approved by MSI’s independent Ethical Review Committee. Local, and national IRB approval was also sought and received in both Kenya and Tanzania as part of their annual patient exit interview survey implementation. Informed consent was sought from all participants immediately prior to the completion of the survey.

## Results

### Provider perspectives

Across all survey timepoints, the majority of the healthcare providers who completed the RMNC training and completed the survey self-identified as being men (64.7% at baseline, 81.0% at 180-days post-training) (see Table 2). About half (47.1%) were midwives and a quarter (23.5%) medical doctors which are in line with the breakdown of provider profiles within MSI Kenya maternities.

**Table 2:** Provider characteristics

	Pre-training (n = 17)	Immediate post-training (n = 13)	90 days post (n = 17)	180 days post (n = 21)	
Gender					
Man	11 (64.7%)	9 (69.2%)	12 (70.6%)	17 (81.0%)	
Woman	6 (35.3%)	4 (30.8%)	5 (29.4%)	3 (14.3%)	
Title					
Nurse*	--	1 (7.7%)	--	1 (4.8%)	
Medical Doctor**	4 (23.5%)	1 (7.7%)	0 (0.0%)	1 (4.8%)	
Midwife	8 (47.1%)	9 (69.2%)	13 (76.5%)	17 (81.0%)	
Anaesthetist	2 (11.8%)	1 (7.7%)	--	--	
Other***	3 (17.6%)	1 (7.7%)	4 (23.5%)	2 (9.5%)	

*Includes Enrolled Nurses (EN) and Registered Clinical Officers (RCO)

**Includes Medical Doctor (MD) and Medical Officer

***Includes Receptionist, Pharmacist, Health Information Records Officer, Sonographer

When asked about their knowledge, attitudes, and perceived or intended behaviours towards RMNC, there was an increase in the proportion of providers who reported stronger agreement with all statements immediately following the provider training (see Tables 3 and 4 and Appendix 1). However, this level of agreement dropped for some statements already at 90 days post-training and for all statements by 180 days post-training. The largest drops in agreement regarding provider behaviours were towards the statements ‘I have a good understanding of childbirth abuse and other types of violence in maternity settings’ (100% of providers strongly agreed with this immediate post-training and 76.2% strongly agreed 180-days post-training), ‘I know how to identify risks or instances of childbirth abuse in my day-to-day work’ (100% vs 80.9%), and ‘I feel confident in calling out instances of childbirth abuse in my workplace’ (100% vs 80.9%). However, the level of strong agreement was still higher at 180 days post-training across all statements than pre-training (Table 3)

Similar patterns were observed for provider’s perceived and intended behaviours, with strong agreement dropping at 180 days post-training compared to immediate post-training but still, levels maintaining a higher proportion of strong agreement than pre-training (Table 4). The strongest drop in the agreement was towards the statement ‘My supervisor(s) support me in delivering respectful maternity care’ (100% strongly agreed immediate post-training compared to 80.9% 180 days post-training)

**Table 3:** Provider attitudes towards RMNC

% of providers who agreed or strongly agreed with each statement	Pre-training (n = 17)	Immediate post (n = 13)	90 days post (n = 17)	180 days post (n = 21)
I have a good understanding of childbirth abuse and other types of violence in maternity settings				
Strongly agree	6 (35.3%)	13 (100%)	17 (100%)	16 (76.2%)
Agree	10 (58.8%)	--	--	5 (23.8%)
I know how to identify risks or instances of childbirth abuse in my day-to-day work				
Strongly agree	7 (41.2%)	13 (100%)	14 (82.4%)	17 (80.9%)
Agree	8 (47.1%)	--	2 (17.7%)	4 (19.1%)
I feel confident in calling out instances of childbirth abuse in my workplace				
Strongly agree	6 (35.3%)	13 (100%)	16 (94.1%)	17 (80.9%)
Agree	8 (47.1%)	--	1 (5.9%)	4 (19.1%)

**Table 4:** Provider perceived and expected RMNC behaviours

% of providers who agreed or strongly agreed with each statement	Pre-training (n = 17)	Immediate post (n = 13)	90 days post (n = 17)	180 days post (n = 21)
My provider colleagues support me in delivering respectful maternity care				
Strongly agree	9 (52.9%)	12 (92.3%)	15 (88.2%)	18 (85.7%)
Agree	7 (41.2%)	1 (7.7%)	2 (11.8%)	3 (14.3%)
My supervisor(s) support me in delivering respectful maternity care				
Strongly agree	10 (58.8%)	12 (92.3%)	15 (88.2%)	17 (80.9%)
Agree	6 (35.3%)	1 (7.7%)	2 (11.8%)	4 (19.1%)
Other providers in my facility provide respectful maternity care				
Strongly agree	8 (47.1%)	8 (61.5%)	15 (88.2%)	19 (90.5%)
Agree	8 (47.1%)	5 (38.5%)	2 (11.8%)	1 (4.8%)

When asked for feedback on the quality of the training immediately post-training, providers reported high satisfaction with the training, with all (100%) of providers in Kenya and Tanzania reporting that they would be likely to recommend the training to other colleagues

### Patient perspectives

In Kenya, of the 156 patients eligible at baseline, 81 (51.9%) calls were made successfully and 47 (58.0%) of these patients completed the survey. At endline, 152 patients were eligible, 130 (85.5%) successfully called and of these 91 (70.0%) completed the survey. A similar range in response rates was seen in Tanzania, with 258 patients eligible at baseline, 238 (92.2%) calls were made successfully and 149 (62.6%) of these patients completed the survey. At endline, 182 patients were eligible, 161 (88.5%) successfully called and of these 80 (49.7%) completed the survey

Across both countries, the majority of patients who completed the questionnaire were ANC patients (Table 5). In Kenya, at baseline two thirds (66.0%) of patients were from the Mombasa maternity while at endline it was closer to a 50/50 split with only 47.3% from the Mombasa maternity and the remaining 52.7% from the Nairobi maternity site

**Table 5:** Patient sample characteristics

	Kenya		Tanzania	
	Baseline	Endline	Baseline	Endline
Total sample (n)	47	91	149	80
Maternity site				
Nairobi	16 (34.0%)	48 (52.7%)	--	--
Mombasa	31 (66.0%)	43 (47.3)	--	--
Dar es Salaam	--	--	149 (100.0%)	80 (100.0%)
Patient category				
ANC	35 (74.5%)	50 (54.9%)	113 (75.8%)	69 (96.3)
Delivery	12 (25.5%)	21 (23.1%)	27 (18.1%)	10 (12.5)
PNC	0 (0.0%)	20 (22.0%)	9 (6.0%)	1 (1.3)

In terms of respectful maternity care outcomes. there was no significant difference in reporting of mistreatment between baseline and endline, as measured by the MIST in either country (Table 6). There was already very low reporting of any mistreatment at baseline, with just 2.0% of patients in Kenya and no (0.0%) patients in Tanzania reporting any instance of mistreatment on the MIST scale

**Table 6:** Mistreatment as measured by the MIST

	Kenya		Tanzania	
	Baseline	Endline	Baseline	Endline
Total sample (n)	47	91	149	80
Reported mistreatment	2 (4.0%)	1 (1.1%)	0 (0.0%)	0 (0.0%)
Reported no mistreatment	45 (96.0%)	90 (98.9)	149 (100.0%)	80 (100.0%)

There was also no significant difference between baseline and endline in experience of respectful maternity care as measured by the MOR scale (Table 7). However, while there was no significant difference in overall MOR scores, significantly more patients disagreed that they felt pushed into accepting options the provider recommended at endline compared to baseline (*p* < 0.01). Additionally, fewer patients disagreed that they were treated poorly due to differences in opinion and patients felt slightly less comfortable asking questions at endline compared to baseline in Kenya (*p* < 0.01). While we saw a similar trends in Tanzania, this result was not significant

**Table 7:** Provision of respect maternity care as measured by the MOR scale

	Kenya		Tanzania	
	Baseline	Endline	Baseline	Endline
Comfortable asking questions	5.9	5.6*	5.7	5.6
Felt pushed into accepting options the provider recommended	3.4	5.1	2.6	4.3*
Treated poorly due to differences in opinion	5.5	5.2*	6.0	5.8
**Overall score**	**76.0**	**75.0**	**77.8**	**78.5**

*Signifies a significant difference between baseline and endline at *p* < 0.0036 following a Bonferroni correction

Regarding patient experience, there were positive trends in patients’ positive agreement with the patient-centred care statements (Table 8). These improvements in agreement to various aspects of patient experience were significant in Tanzania for a subset of areas of care, including providers doing a better job of explaining what they were doing (Tanzania, *p* < 0.05), patient choices being respected by all staff (*p* < 0.01), patients being satisfied with their birth plan (*p* < 0.05), patients being satisfied with pain management offered (*p* < 0.01), patients having enough privacy (*p* < 0.01). While the differences in agreement to these statements could not be reliably tested in the Kenya patient data due to categories having too few responses, we did see similar trends in a higher proportion of patients reporting that they strongly agreed that these aspects of care were achieved at endline as compared to baseline

**Table 8:** Patient experience by country

		Kenya									Tanzania								
	Timeframe	Yes		No	DK		% yes			p-value	Yes	No		DK		% yes			p-value
Provider explained options for birth plans, pain management, and birth partners	Baseline	37		8	2		78.7			**0.877**	147	2		0		99%			**1.000**
	Endline	67		20	4		73.6				79	1		0		99%			
Allowed to move around during labour	Baseline	8		4	0		66.7			na	36	0		0		100%			1.000
	Endline	39		2	0		95.1				11	0		0		100%			
Companion was allowed	Baseline	9		3	0		75.0			na	35	0		1		97%			1.000
	Endline	**39**		**1**	**1**		95.1				11	0		0		100%			
		**Kenya**									**Tanzania**								
	**Timeframe**	**SD**	**D**	**N**	**A**	**SA**		**% SA**	**p-value**		**SD**	**D**	**N**		**A**		**SA**	**% SA**	**p-value**
Providers spoke in an understandable manner	Baseline	0	0	0	2	45		95.7	**na**		0	0	0		62		87	58.4	**0.113**
	Endline	1	0	1	1	89		97.8			0	0	0		24		56	70.0	
Providers explained what they were doing	Baseline	0	0	0	4	43		91.5	**na**		0	0	0		59		90	60.4	0.038*
	Endline	1	1	0	2	87		95.6			0	0	0		20		60	75.0	
Provider took time to listen to questions	Baseline	0	0	0	3	44		93.6	**na**		0	1	0		58		90	60.4	**na**
	Endline	0	0	1	2	89		97.8			0	0	0		18		62	77.5	
Trusted providers and other staff	Baseline	0	0	2	5	40		85.1	**na**		0	0	1		61		87	58.4	**na**
	Endline	1	0	1	1	88		96.7			0	0	0		20		60	75.0	
Choices were respected by all staff	Baseline	0	0	0	3	3		75.0	**na**		0	0	0		23		13	36.1	0.001**
	Endline	0	0	1	0	40		97.6			0	0	0		0		11	100	
Satisfied with birth plan	Baseline	2	5	0	13	15		42.9	**na**		0	0	7		40		66	58.4	0.013*
	Endline	4	5	13	4	24		48.0			1	0	0		15		53	76.8	
Satisfied with pain management offered	Baseline	0	0	2	1	9		75.0	na		1	0	0		24		11	30.6	0.000**
	Endline	0	0	1	0	40		95.1			0	0	0		0		11	100	
Had enough privacy	Baseline	0	0	0	2	10		83.3	na		0	0	0		23		13	36.1	0.001**
	Endline	1	0	0	1	39		97.6			0	0	0		0		11	100	

SA = Strongly Agree, A = Agree, N = Neutral, D = Disagree, SD = Strongly Disagree, DK = Don’t Know

*Signifies a significant difference between baseline and endline at *p* < 0.05

**Signifies a significant difference between baseline and endline at *p* < 0.01

na = Matrices evaluated with chi-square tests were collapsed for categories without counts in the baseline and endline datasets. Chi-squared test values were not considered reliable if either at least one expected count is 0 or 20% or more of the expected counts are less than 5

## Discussion

This study describes healthcare providers’ perceived knowledge, attitudes and expected behaviours before and after they received the RMNC training as well as the perception of patients regarding respectful care before and after the provider training was rolled out in their maternity facility

The patient-centred questionnaire from MSI is similar to the Mother’s Autonomy in Decision Making (MADM) scale [22]. Results showed that almost all patients (> 95%) were encouraged and allowed to always move and have a birth companion with them during labour. The sample was too small to show statistical significance, but we expect that with a better powered sample we could confirm the significance of this trend. A similar evaluation with an appropriately powered sample is currently underway in Ethiopia to confirm this hypothesis

The patient-centred care indicators allowed us to explore additional aspects of care and we saw positive trends in the proportion of patients reporting that they strongly agreed that providers were more approachable, including explaining better what care they were providing (*p* < 0.05, Tanzania) in an understandable manner. Patients in Tanzania were also more likely to report that their choices were respected by staff (*p* < 0.01) 6-months following the training, that they were satisfied with their birth plan (*p* < 0.05), that they were satisfied with the pain management offered (*p* < 0.01) and that they had enough privacy (*p* < 0.01). Similar trends were seen in Kenya, indicating that the training supported the providers to offer more respectful, patient-centred care. It is possible as well that the training has supported broader behaviour change and reinforcement of structural change in the 3 maternities

When applying the MOR index [23], the change in the overall score was not statistically significant between baseline and endline. However, when assessing the unique categories of the MOR scale, fewer patients disagreed with feeling pushed into accepting options, indicating positive shifts in the provision of respectful care. We did also find that more patients disagreed that they were treated poorly due to differences in opinion and patients felt slightly less comfortable asking questions at endline compared to baseline in Kenya (*p* < 0.01). However, the scores were still high at above 5.5 with a score of 6 being the maximum. This slight difference may not represent a concern even if significant

The MIST index [24] was applied to make sure that any kind of mistreatment was identified and ruled out at baseline and endline. Both base and endline showed extremely positive results of 96% or higher, excluding blunt mistreatment forms in all but a significant minority patients interviewed

These results underline several key issues. We had invested in making sure all physical resources were supporting RMNC in all maternities and we had worked on making sure protocols and resources were focusing on respectful care and positive patient experience. This was, however, not enough to ensure respectful practices on a routine basis for all patients, such as labour companions, different labour positions offered and respected or routine skin-to-skin practised after delivery. Only after implementing the workshop, these obstetric practices were perceived as changing

These results are aligned with previous findings from an evaluation of a respectful maternity care intervention in Ethiopian hospitals including provider training with a behavioural change component, which found a significantly fewer mistreatment experiences reported during childbirth following implementation of the intervention [25]

This is underpinned by the results from providers’ surveys. There was a clear and drastic improvement in knowledge of RMNC (e.g., ability to identify forms of obstetric violence) from before to right after having received the training. The same questions however showed a decrease in knowledge after 3 months and a further decrease after 6 months

A similar trend was seen when questioning perceived and intended RMNC practices by the provider itself but also by the team and supervisor (e.g., felt supervisor and fellow colleagues understand and provide respectful practices). The immediate before and after showed remarkable improvements. However, the perceived practices decreased after 3 and 6 months. The decrease was less marked than the one of the perceived knowledge areas

Both decreases in perceived knowledge and practices were expected as similar studies had already shown the same dose and time effect of provided information [26]. It clearly underlines the need for strategies to keep RMNC alive in all facilities and for all providers, for example, through frequent, light-dose refresher trainings.

Not less important was the high satisfaction expressed by all providers that received the training package. The workshop is highly participative, and high satisfaction supports further work on RMNC.

Currently, the same roll-out is taking place in the 10 MSI Ethiopian maternities with much higher patient volumes that will allow for a more robust evaluation. The presented data are only the start, and the Ethiopia experience will shed further light on how changes in obstetric practices are fostered by behaviour and knowledge change and a supportive organisational culture.

### Limitations and strengths of the study

As detailed in the background, the training toolkit was rolled out along with various organisational and facility-based improvements to processes, systems, and infrastructures to complement and support the provision of respectful care. This means that possible effects of the training may have been confounded or mediated by other processes that we could not control for in this study.

We were also limited in our analysis of the provider data due to a limited sample size in Kenya and missing data at two time points in Tanzania. Given the provider characteristics (gender and title) at the various time points, it is also possible providers who did not participate in the in-person training completed the endline survey, explaining the shift in these characteristics. This could be due to confusion between the online module and the in-person training. Further, as no unique IDs were used to support anonymity, we could not match between the survey timepoints to distinguish who from the baseline survey completed all survey timepoints. This will be addressed in the Ethiopia study to ensure possibility of matching respondents between surveys.

Another limitation in our analysis was that high baseline scores in the patient survey limited our power to detect any change in endline. This could be explained by low awareness of what standard of care to expect or that the data collection tools were not fit for purpose. We also did not conduct the survey pre-development of RMNC package so the tools were not specifically adapted to focus on the any change in specific areas of disrespectful or abusive care that may have been identified by such formative research.

To delve deeper into quality of respectful care as measure by the MOR scale, we assessed differences in scores by each category of the MOR. However, individual variables from the MOR scale have not been validated on their own to our knowledge so these results should be interpreted cautiously.

Another possible limitation of our patient data analysis was the lower response rate than predicted. We gave the option for patients to request a call back time when first contacted by phone. However, many patients did not answer the phone when called back. Many calls were also answered by someone other than the patient. This led to response rates around 50% and we have no way of knowing what profile of patients were excluded from our final sample.

Furthermore, given that data collectors were contact centre agents employed by the same organisation that manages the maternity hospitals, there is a risk of underreporting results perceived to be reputationally damaging.

Lastly, neither the results from the patient survey nor the provider survey can be extrapolated to other populations or geographies.

While it is important to consider the limitations when interpreting the results, there are also key strengths of the study including that both provider and patient perspectives were captured. The study also drew on 3 different validated patient questionnaires. Lastly, we were able to assess provider perspectives at multiple time-points (3 and 6-months following the training) and not just immediately following the training to assess the sustainability of any effects from the training.

## Conclusions

This study demonstrates that healthcare worker knowledge, attitudes and RMNC practices can be improved with this training intervention. The findings also suggest that patients report a more positive experience of their maternity care after the training was implemented.

We believe that structural, physical, and organizational changes support RMNC, without a complete change in provider beliefs and attitudes towards deeply rooted obstetric practices, the change towards routine respectful care for all patients will not happen

We saw that the effects of the training on provider knowledge, attitudes and perceived or intended behaviours may decrease over time. Therefore, the effect of the training toolkit must be further sustained through a variety of strategies such as routine refresher trainings, supportive supervision, appointment of RMNC champions in each maternity and integration of RMNC micro-activities in monthly staff meetings

However, this evaluation demonstrates that changes in obstetric practices such as birth companions were not successfully implemented until they came directly from the knowledge and conviction of the providers and not through an external imposition or by improving physical resources

A combination of physical and staffing resources as well as behaviour change methodologies are needed hand-in-hand for a long-lasting, sustainable change towards respectful maternity care

### Electronic supplementary material

Below is the link to the electronic supplementary material.

Supplementary Material 1

## Data Availability

The datasets used and/or analysed during the current study are available from the corresponding author upon reasonable request.

## Footnotes

1 In this manuscript, we will use the gender-inclusive terms ‘pregnant person’, ‘people’ or ‘individuals’ to refer to those who are capable of pregnancy, as not all people who get pregnant or seek pregnancy care identify as women. We will use gender explicit (ex:. ‘women’) when referring to prior research that describes study subjects as such.

2 Verbal abuse: Harsh or rude language; shouting, insults, scolding, mocking; judgemental or accusatory comments; threats of withholding treatment or of using unnecessary treatment, or of poor outcomes; blaming for current situation, or current or potential future poor outcomes. Physical abuse: Being beaten, slapped, kicked, punched, or pinched; physical restraint; gagged; physically tied down; (childbearing women: forceful downward pressure); rough use of instruments or interventions.” (14).

## Abbreviations

ANC: Antenatal Care
CAPI: Computer-Assisted Personal Interviewing
CPD: Continuing Professional Development
MADM: Mother?s Autonomy in Decision Making
MIST: Mistreatment Index
MOR: Mothers on Respect
PNC: Postnatal care
PTSD: Post-Traumatic Stress Disorder
RMNC: Respectful Maternity and Neonatal Care
SRH: Sexual and Reproductive Health
ToT: Training of Trainers
VCAT: Values Clarification and Attitudes Transformation
WHO: World Health Organisation
