# Supplementary material for: When addressing resources is not enough: lessons learned from a respectful maternal and neonatal care provider training intervention evaluation in Kenya and Tanzania
Source: BMC Pregnancy Childbirth. 2024 May 14;24:359. doi: 10.1186/s12884-024-06555-3 (PMC11094886; doi:10.1186/s12884-024-06555-3)
Supplement: Supplementary file 1 — Supplementary Material 1 [file 12884_2024_6555_MOESM1_ESM.docx]

## Supplementary Material

### Appendix 1a-b: Complete table of provider knowledge, attitudes, and practices (Kenya only)

| ***(1a) Knowledge & attitudes*** | **Pre-training**  (n=17) | | | **Post-training** (n=13) | | **3-month post** (n=17) | | **6-month post**  (n=21) | |
| --- | --- | --- | --- | --- | --- | --- | --- | --- | --- |
|  | No. | row % | | No. | row % | No. | row % | No. | row % |
| **I have a good understanding of childbirth abuse and other types of violence in maternity settings** |  |  | |  |  |  |  |  |  |
| Strongly agree | 6 | 35.3 | | 13 | 100 | 17 | 100 | 16 | 76.2 |
| Agree | 10 | 58.8 | |  |  |  |  | 5 | 23.8 |
| Disagree |  |  | |  |  |  |  |  |  |
| Strongly disagree |  |  | |  |  |  |  |  |  |
| Don't know | 1 | 5.9 | |  |  |  |  |  |  |
| **I feel confident in my abilities to assist a patient with care and respect during delivery** |  |  | |  |  |  |  |  |  |
| Strongly agree | 9 | 52.9 | | 13 | 100 | 17 | 100 | 18 | 85.7 |
| Agree | 6 | 35.3 | |  |  |  |  | 3 | 14.3 |
| Disagree | 1 | 5.9 | |  |  |  |  |  |  |
| Strongly disagree |  |  | |  |  |  |  |  |  |
| Don't know | 1 | 5.9 | |  |  |  |  |  |  |
| **I can easily understand patient’s fears and worries about pregnancy and childbirth** |  |  | |  |  |  |  |  |  |
| Strongly agree | 5 | 29.4 | | 13 | 100 | 16 | 94.1 | 17 | 80.9 |
| Agree | 11 | 64.7 | |  |  | 1 | 5.9 | 4 | 19.1 |
| Disagree | 1 | 5.9 | |  |  |  |  |  |  |
| Strongly disagree |  |  | |  |  |  |  |  |  |
| Don't know |  |  | |  |  |  |  |  |  |
| **I know how to identify risks or instances of childbirth abuse in my day-to-day work** |  |  | |  |  |  |  |  |  |
| Strongly agree | 7 | 41.2 | | 13 | 100 | 14 | 82.4 | 17 | 80.9 |
| Agree | 8 | 47.1 | |  |  | 3 | 17.7 | 4 | 19.1 |
| Disagree | 1 | 5.9 | |  |  |  |  |  |  |
| Strongly disagree |  |  | |  |  |  |  |  |  |
| Don't know | 1 | 5.9 | |  |  |  |  |  |  |
| **I feel confident in calling out instances of childbirth abuse in my workplace** |  |  | |  |  |  |  |  |  |
| Strongly agree | 6 | 35.3 | | 13 | 100 | 16 | 94.1 | 17 | 80.9 |
| Agree | 8 | 47.1 | |  |  | 1 | 5.9 | 4 | 19.1 |
| Disagree | 2 | 11.8 | |  |  |  |  |  |  |
| Strongly disagree |  |  | |  |  |  |  |  |  |
| Don't know | 1 | 5.9 | |  |  |  |  |  |  |
| **My colleagues believe that yelling at or scolding a patient is never acceptable** |  |  | |  |  |  |  |  |  |
| Strongly agree | 11 | 64.7 | | 13 | 100 | 14 | 82.4 | 18 | 85.7 |
| Agree | 5 | 29.4 | |  |  | 2 | 11.7 | 3 | 14.3 |
| Disagree | 1 | 5.9 | |  |  |  |  |  |  |
| Strongly disagree |  |  | |  |  | 1 | 5.9 |  |  |
| Don't know |  |  | |  |  |  |  |  |  |
|  |  |  | |  |  |  |  |  |  |
| ***(1b) Expected or perceived practices*** | **Pre-training**  (n=17) | | | **Post-training** (n=13) | | **3-month post** (n=17) | | **6-month post**  (n=21) | |
|  | No. | row % | | No. | row % | No. | row % | No. | row % |
| **My provider colleagues support me in delivering respectful maternity care** |  |  | |  |  |  |  |  |  |
| Strongly agree | 9 | 52.9 | | 12 | 92.3 | 15 | 88.2 | 18 | 85.7 |
| Agree | 7 | 41.2 | | 1 | 7.7 | 2 | 11.8 | 3 | 14.3 |
| Disagree | 1 | 5.9 | |  |  |  |  |  |  |
| Strongly disagree |  |  | |  |  |  |  |  |  |
| Don't know |  |  | |  |  |  |  |  |  |
| **My supervisor(s) support me in delivering respectful maternity care** | | |  |  |  |  |  |  |  |
| Strongly agree | 10 | 58.8 | | 12 | 92.3 | 15 | 88.2 | 17 | 80.9 |
| Agree | 6 | 35.3 | | 1 | 7.7 | 2 | 11.8 | 4 | 19.1 |
| Disagree |  |  | |  |  |  |  |  |  |
| Strongly disagree |  |  | |  |  |  |  |  |  |
| Don't know | 1 | 5.9 | |  |  |  |  |  |  |
| **Other providers in my facility provide respectful maternity care** |  |  | |  |  |  |  |  |  |
| Strongly agree | 8 | 47.1 | | 8 | 61.5 | 15 | 88.2 | 19 | 90.5 |
| Agree | 8 | 47.1 | | 5 | 38.5 | 2 | 11.8 | 1 | 4.8 |
| Disagree |  |  | |  |  |  |  | 1 | 4.8 |
| Strongly disagree | 1 | 5.9 | |  |  |  |  |  |  |
| Don't know |  |  | |  |  |  |  |  |  |
| **I feel confident that I will discuss respectful maternity care with friends or family** |  |  | |  |  |  |  |  |  |
| Strongly agree |  |  | | 13 | 100 | 15 | 88.0 | 19 | 90.0 |
| Agree |  |  | |  |  | 2 | 12.0 | 1 | 5.0 |
| Disagree |  |  | |  |  |  |  |  | 0 |
| Strongly disagree |  |  | |  |  |  |  | 1 | 5.0 |
| Don't know |  |  | |  |  |  |  |  |  |

### Appendix 2: Complete analysis of MOR categories by country and pooled sample

|  | **Kenya** | | **Tanzania** | |
| --- | --- | --- | --- | --- |
|  | **Baseline** | **Endline** | **Baseline** | **Endline** |
| **Comfortable asking questions** | 5.9 | 5.6* | 5.7 | 5.6 |
| **Comfortable declining care** | 5.8 | 5.6 | 5.6 | 5.6 |
| **Comfortable accepting care options the provider recommended** | 5.8 | 5.6 | 5.6 | 5.6 |
| **Felt pushed into accepting options the provider recommended** | 3.4 | 5.1 | 2.6 | 4.3* |
| **Chose the care options received** | 5.7 | 5.5 | 5.6 | 5.6 |
| **Personal preferences were respected** | 5.8 | 5.5* | 5.6 | 5.6 |
| **Cultural preferences were respected** | 5.7 | 5.6 | 5.6 | 5.5 |
| **Treated poorly due to race, ethnicity, cultural background, or language** | 5.5 | 5.2 | 6.0 | 5.9 |
| **Treated poorly due to sexual orientation or gender** | 5.5 | 5.2 | 6.0 | 5.8 |
| **Treated poorly due to health insurance** | 5.5 | 5.2 | 6.0 | 5.8 |
| **Treated poorly due to differences in opinion** | 5.5 | 5.2 | 6.0 | 5.8 |
| **Held back from asking questions as provider was rushed** | 5.4 | 5.2 | 5.9 | 5.8 |
| **Held back from asking as care wanted differed from provider** | 5.4 | 5.2 | 5.9 | 5.9 |
| **Held back from asking questions as did not want to seem difficult** | 5.5 | 5.2 | 5.9 | 5.9 |
| **Overall score** | **76.0** | **75.0** | **77.8** | **78.5** |

*Signifies a significant difference between baseline and endline at p < 0.0036 following a Bonferroni correction.

### Appendix 3. Complete patient questionnaire

| **#** | **Question text** | **Response options** | | | | | | | **Skip pattern** | **Variable name** | |
| --- | --- | --- | --- | --- | --- | --- | --- | --- | --- | --- | --- |
| **1. Interview and site information (enter questions 1-4 before starting the call)** | | | | | | | | | | | |
| 1 | Today's date | __/ __/ ____ | | | | | | | All | DATE | |
| 2 | Select the centre where the patient received their service | Eastleigh Nursing Home (1)  Kumasi Nursing Home (2)  Mwenge Hospital (3)  Mombasa Nursing Home (4) | | | | | | | All | SITE | |
| 3 | Select the patient type | ANC (1)  Delivery (2)  PNC (3) | | | | | | | All | TYPE | |
| 4 | Card number (please insert the card number as written in the call back list) | Enter card number: __________  Re-enter card number: ___________ | | | | | | | All |  | |
| 5 | Patient consented to complete the questionnaire | Yes (1) No (0) If NO, end call | | | | | | | All | CONSENT | |
| 8 | Did the patient agree that their data could be shared with other researchers? | Yes (1) No (0) | | | | | | | All | DATAUSE | |
| **2. Patient Experience** | | | | | | | | | | | |
| 1 | How likely are you to recommend our services to other mothers or expectant mothers? | Not at all likely (0)  …  Very likely (10) | | | | | | | Ask all | REC | |
| 2 | Based on your most recent experience, how likely is it that you would return to Marie Stopes in the future if you needed to deliver again? | Not at all likely (0)  …  Very likely (10) | | | | | | | Delivery & PNC only | RET | |
| 3 | Overall, how satisfied are you with your experience at Marie Stopes? | Completely satisfied (1)  Satisfied (2)  Neither satisfied nor dissatisfied (3)  A bit dissatisfied (4)  Not at all satisfied (5) | | | | | | | Ask all | SAT | |
| ***Read to respondent: For the following questions, please rate how strongly you agree or disagree with the following statement from 1 to 5, with 5 being strongly agree.*** | | | | | | | | | | | |
| 4 | The healthcare providers or other staff at the facility spoke to you in a language you could understand.  *Healthcare providers refer to doctors, nurses, or midwives that you may have interacted with.* | Strongly agree (1)  Agree (2)  Neither agree nor disagree (3)  Disagree (4)  Strongly disagree (5) | | | | | | | Ask all | UNDERSTSAND | |
| 5 | The healthcare providers explained to you why and how they were doing examinations or procedures on you, or why they were giving you medications | Strongly agree (1)  Agree (2)  Neither agree nor disagree (3)  Disagree (4)  Strongly disagree (5) | | | | | | | Ask all | EXPLAIN | |
| 6 | The healthcare provider took time to listen to your questions and concerns | Strongly agree (1)  Agree (2)  Neither agree nor disagree (3)  Disagree (4)  Strongly disagree (5) | | | | | | | Ask all | LISTEN | |
| 7 | You were always treated with respect by all staff members | Strongly agree (1)  Agree (2)  Neither agree nor disagree (3)  Disagree (4)  Strongly disagree (5) | | | | | | | Ask all | RESPECT | |
| 9 | The provider clearly explained options to you for birth positions, birth partners, and pain management | Yes (1) No (0)  Don’t know (999) | | | | | | | Ask all | OPTIONS | |
| 9a | You are satisfied with the birth plan for your delivery.  *A birth plan is an outline of your preferences during your labour and delivery* | Strongly agree (1)  Agree (2)  Neither agree nor disagree (3)  Disagree (4)  Strongly disagree (5) | | | | | | | ANC patients only | PLAN | |
| 9b | Did you have a birth plan in place when you were admitted?  *A birth plan is an outline of your preferences during your labour and delivery* | Yes (1) No (0)  Don’t know (999) | | | | | | | Delivery & PNC patients | PLAN1 | |
| 9c | The provider took the time to review your birth plan when you were admitted | Yes (1) No (0)  Don’t know (999) | | | | | | | 12a = YES | PLAN2 | |
| 11a | I was offered pain management options and provided these when requested (i.e., pain relief offered during labour or anaesthesia during stitching) | Yes (1) No (0)  Don’t know (999) | | | | | | | Delivery & PNC patients | PAIN1 | |
| 11b | I was happy with the pain management options offered | Strongly agree (1)  Agree (2)  Neither agree nor disagree (3)  Disagree (4)  Strongly disagree (5) | | | | | | | Delivery & PNC patients | PAIN2 | |
| 12 | I feel like I had enough privacy during labour and delivery | Strongly agree (1)  Agree (2)  Neither agree nor disagree (3)  Disagree (4)  Strongly disagree (5) | | | | | | | Delivery & PNC patients | PRIV | |
| 13 | I feel that the choices I made for my delivery were respected by all staff members | Strongly agree (1)  Agree (2)  Neither agree nor disagree (3)  Disagree (4)  Strongly disagree (5) | | | | | | | Delivery & PNC patients | CHOICES | |
| 14 | Your baby always stayed with you, unless there was a medical issue | Yes (1) No (0)  Don’t know (999) | | | | | | | Delivery & PNC patients | SEPERATE | |
| 15 | The provider checked regularly on how you were feeling after your delivery | Strongly agree (1)  Agree (2)  Neither agree nor disagree (3)  Disagree (4)  Strongly disagree (5) | | | | | | | PNC patients only | CHECK | |
| 16 | You felt adequately supported by your healthcare provider not only in your physical health but also mental health during the postpartum period | Strongly agree (1)  Agree (2)  Neither agree nor disagree (3)  Disagree (4)  Strongly disagree (5) | | | | | | | PNC patients only | MENTAL | |
| **3. Facility environment and culture** | | | | | | | | | | | |
| 1 | Were you allowed food and drink during labour if no medical complications limited this? | Yes (1) No (0)  Don’t know (999) | | | | | | | Delivery & PNC patients | FOOD | |
| 2 | Were you allowed to move around during labour if you wished to, if no medical complications limited this? | Yes (1) No (0)  Don’t know (999) | | | | | | | Delivery & PNC patients | MOVE | |
| 3 | Were you allowed to have someone you wanted (outside of staff at the facility, such as family or friends) to stay with you during labour and delivery? | Yes (1) No (0)  Don’t know (999) | | | | | | | Delivery & PNC patients | COMPAN | |
| 4 | Did you experience any of the following problems or attitudes in your care during pregnancy, birth, or post-natal care?  *Please select all applicable options* | Your private or personal information was shared without your consent (1) Your physical privacy was violated, for example being uncovered or having people in the delivery room without your consent (2)  A healthcare provider shouted at or scolded you (3)  Healthcare providers withheld treatment or forced you to accept treatment that you did not want (4)  Healthcare providers threatened you in any way (5)  Healthcare providers ignored you, refused your request for help or failed to respond to requests for help in a reasonable amount of time (6)  You experienced physical abuse, such as aggressive physical contact, inappropriate sexual conduct, etc. (7)  None of the above (8) | | | | | | | Ask all | MIST | |
| 5 | During your time at the facility, did any staff at the facility ask you or your family for money other than the official cost of service to access services or favors? | Yes (1) No (0)  Don’t know (999) | | | | | | | Ask all | BRIBE | |
| 6 | Do you feel like your health information was, or will be, kept confidential at the Marie Stopes maternity? | Strongly agree (1)  Agree (2)  Neither agree nor disagree (3)  Disagree (4)  Strongly disagree (5) | | | | | | | Ask all | INFO | |
| **4. Delivery of respectful care (MOR: Mothers on respect Index)** | | | | | | | | | | | |
| A | **Overall while making decisions about my pregnancy or birth care:** (Select one) | Strongly disagree (1) | Disagree | Somewhat disagree | Somewhat agree | Agree | Strongly agree (6) | Ask all | | | MOR_A |
|  | I felt comfortable asking questions |  |  |  |  |  |  |  |  |  |  |
|  | I felt comfortable declining care that was offered |  |  |  |  |  |  |  |  |  |  |
|  | I felt comfortable accepting the options for care that my healthcare provider recommended |  |  |  |  |  |  |  |  |  |  |
|  | I felt pushed into accepting the options my healthcare provider suggested* |  |  |  |  |  |  |  |  |  |  |
|  | I chose the care options that I received |  |  |  |  |  |  |  |  |  |  |
|  | My personal preferences were respected |  |  |  |  |  |  |  |  |  |  |
|  | My cultural preferences were respected |  |  |  |  |  |  |  |  |  |  |
| B | **During my pregnancy I felt that I was treated poorly by my healthcare provider because of:** (Select one) | Strongly disagree (6) | Disagree | Somewhat disagree | Somewhat agree | Agree | Strongly agree (1) | Ask all | | | MOR_B |
|  | My race, ethnicity, cultural background, or language* |  |  |  |  |  |  |  |  |  |  |
|  | My sexual orientation and / or gender identity* |  |  |  |  |  |  |  |  |  |  |
|  | My type of health insurance or lack of insurance* |  |  |  |  |  |  |  |  |  |  |
|  | A difference of opinion with my caregivers about the right care for myself or my baby* |  |  |  |  |  |  |  |  |  |  |
| C | **During my pregnancy I held back from asking questions or discussing my concerns because:** (Select one) | Strongly disagree (6) | Disagree | Somewhat disagree | Somewhat agree | Agree | Strongly agree (1) | Ask all | | | MOR_C |
|  | My healthcare provider seemed rushed* |  |  |  |  |  |  |  |  |  |  |
|  | I wanted maternity care that differed from what my healthcare provider recommended* |  |  |  |  |  |  |  |  |  |  |
|  | I thought my healthcare provider might think that I was being difficult* |  |  |  |  |  |  |  |  |  |  |
